# Digital vectors of disease: how alcohol ‘smart’ labels leapfrog health policy

**DOI:** 10.1186/s12889-026-27456-4

**Published:** 2026-05-29

**Authors:** Anna Coghlan, Norah Campbell, Amber van Den Akker

**Affiliations:** 1 Trinity Business School, Trinity College Dublin, Dublin, D02 F6N2 Ireland; 2 Department for Health, University of Bath, Claverton Down , Bath, BA2 7AY England

**Keywords:** Commercial Determinants of Health, Alcohol policy, Labelling, Digital vectors of disease

## Abstract

**Background:**

The use of digital technologies by unhealthy commodity industries (UCIs) warrants closer investigation as a potential vector of disease. Examining one such digital technology, this research analyses the use of “smart labels” by the alcohol industry (labels which have physical and digital dimensions which can sense, process, and deliver real time and immersive information and entertainment between product and consumer) to investigate if rapidly advancing technologies create a gap between policy and technological innovation in general, and examine whether smart labels in the alcohol industry present a threat to public health in particular.

**Methods:**

This study analyses alcohol brands’ smart label use within the ten largest alcohol companies operating in Ireland since 2010. Using framing analysis to examine smart labels used by different alcohol brands, we sought to address three questions: (i) what are the technological capacities of smart labels? (ii) what justifications does the alcohol industry use to normalise smart labels? (iii) how does this technology impact health when considered through a the lens of commercial determinants of health?

**Results:**

Smart labelling has been justified by the alcohol industry as a technology that creates a more informed consumer, enhances corporate social responsibility and regulatory compliance, assures product quality, and leads to more responsible consumption. We examine these frames by introducing the MORAL framework. To contrast, we consider the impact of smart labels on public health through a commercial determinants of health perspective, showing that smart labelling (i) habituates consumption, (ii) undermines health information presented on physical labels, (iii) drives the next revolution in the micro-targeting of consumers, and (iv) is rapidly establishing an industry norm, in order to leapfrog current regulation. We articulate this through a HARM framework.

**Conclusions:**

Smart labelling is a novel commercial determinant of health. Through rapid innovation of traditional labelling practices, the alcohol industry has leveraged smart labels to leapfrog regulation and perpetuate patterns of harm to public health. Our findings underscore the expanded playbook capabilities of the alcohol industry to outpace policy in the digital age, and suggest that it is crucial for policy to either rapidly evolve, or to perpetually remain one step behind unhealthy commodity industries.

## Background

### Digital innovations as commercial vectors of disease

Commercial actors profoundly shape the environments in which populations live and work. While some benefit health, others have been known to drive systems of health-harm and expedite rates of non-communicable diseases including cancer, cardiovascular disease, diabetes, and respiratory disease [1, 2] in pursuit of market growth. 18% of annual mortality in the WHO European Region is caused by just three unhealthy commodity industries: alcohol, ultra-processed foods, and tobacco [1]. These industries have been classified as “unhealthy commodity industries” (UCIs): industries which cause adverse health impacts via the production of unhealthy commodities and the related commercial activities and strategies used to promote their consumption [3]. The commercial determinants of health (CDoH) as a concept is increasingly used in both research and policy to capture the systems, practices, and pathways through which industries shape public health and influence the policy landscape [4, 5].

Across unhealthy commodity industries (UCIs), digital innovation has led to the introduction of “smart” labels – digital tags embedded into products which can sense, process, and deliver real-time, immersive information and entertainment between product and consumer using smartphones [6, 7]. These innovations rapidly change the way the industries conducts business, and challenge the regulatory bodies charged with protecting population health against the effects of these industries [8–11].

### The policy-digitalisation gap

The imperative for market expansion and year-on-year increases in revenue demands successive technological innovation cycles by UCIs. The gambling industry, for example, continuously develops new generations of electronic-gambling machines and electronic table-games [12]; the ultra-processed food industry leverages collaborations with digital platforms to integrate personalised marketing seamlessly into consumers’ lives [1]; the tobacco industry develops technological capacities within electronic cigarettes, such as personal vaporisers and larger heating coils [1, 13]. In a context characterised by rapid technological innovation and generational leaps in capability, the current pace of policymaking may struggle to keep pace with innovation – leading to a policy-digitalisation gap. Although terminology varies, this ‘gap’ has been described across disciplines: The Collingridge dilemma states that while regulation of new technologies is possible, it is often delayed until the technology is too entrenched to control easily [14]; the innovation-policy gap within political science tracks how the digital economy outpaces regulatory processes [15]; and the pacing problem in bioethics has shown that technologies advance exponentially, while policy follows incremental and reactive trajectories [16]. Exponential, iterative, and breakthrough technology innovations overwhelm the incrementalist and often reactive policy process [15, 16]. For example, while e-cigarette technology entered the mainstream in 2010 [17], it was another 11 years before the Irish Public-health Tobacco Act was updated to cover the definition of “tobacco product” and “electronic cigarette” [18]. The Public Health (Alcohol) Act, introduced into the Irish parliament in 2015, will not come into full effect until 2028 at the earliest – a 13 year span between introduction and full enactment [19–21]. This policy-digitisation gap shows the additional advantage of delay: as a window of opportunity to embed technological innovations that leapfrogs legislation.

The transnational commercial organisations driving innovation become the gatekeepers of innovation, leading to governments relying on UCIs to give them advice and knowledge on the technologies [22]. In addition to this relationship of reliance, it has been argued that UCI involvement in policy discussions can lead to an overly-narrow lens on the technology under consideration; countering and outweighing alternatives, and promoting the belief that the latest technology developed by the industry itself offers the best public health solutions [23, 24].

Future-proofed legislation can bridge the policy-digitalisation gap. For example, the Standardised Packaging of Tobacco Act in Ireland (2015) demonstrated policy foresight, broadening bar-code[s] to include *“similar identification mark[s]* printed on the outside packaging” [25] – a definition which encompassed QR-codes indirectly. However, technology has since evolved, and sometimes, printed codes are not required. For example, Dunhill British-American tobacco packets contained small digital stickers called NFC (near-field communication) tags so that, when its plain packaging was tapped with a phone, the consumer was led to exclusive content and rewards online [26]. This illustrates the challenges that policymakers are grappling with in rapid technology arenas.

**Fig. 1:**
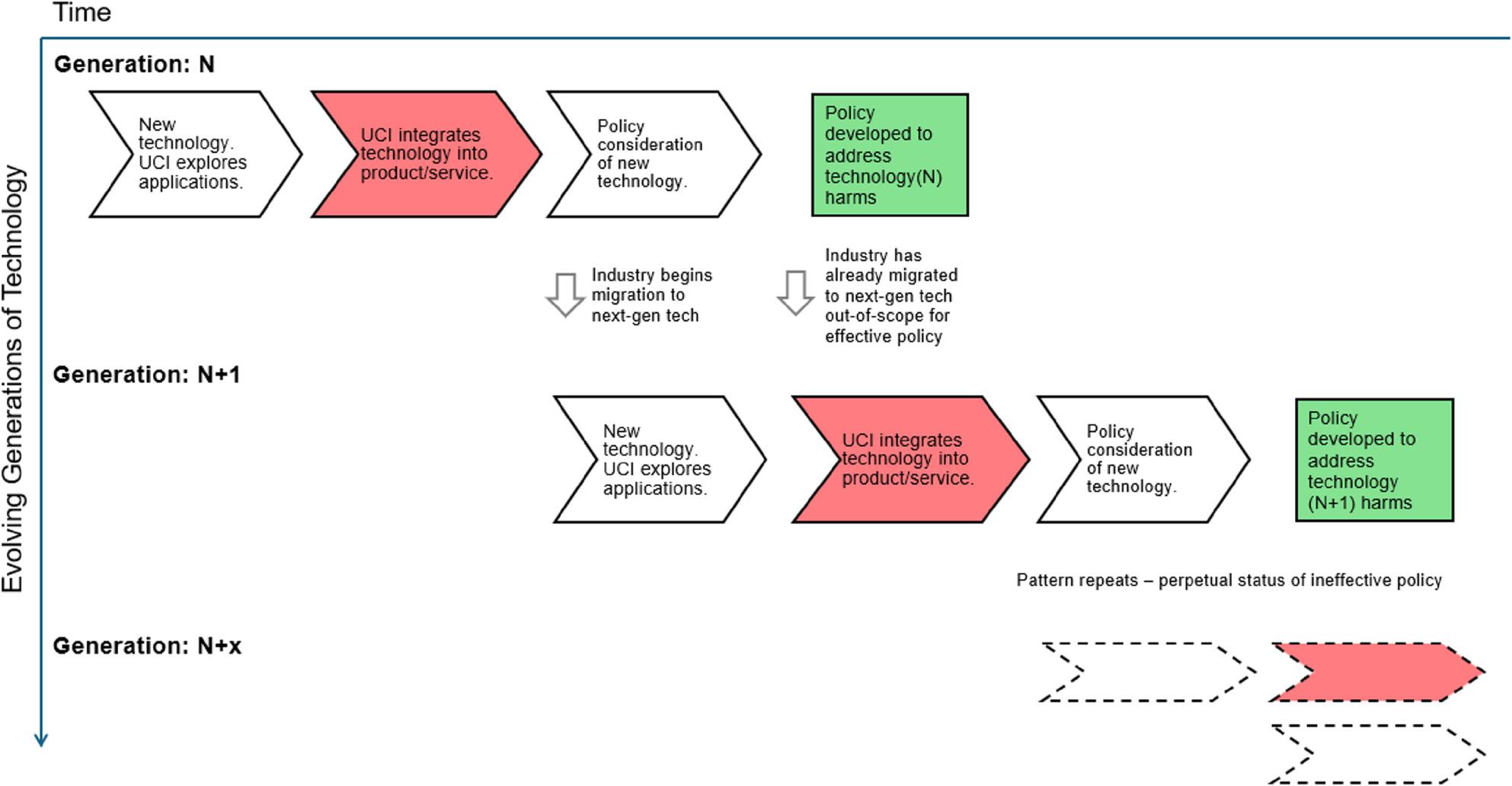
The policy-digitalisation gap

This dynamic shows how, in parallel with efforts to both delay and narrow policy, UCIs simultaneously innovate to the next generation of technology – one which is designed to be beyond the scope of in-progress legislation (see Fig. 1). To examine the impact of policy-digitalisation gaps on regulation to address harmful industries’ impact on health, we analyse alcohol brands’ smart label use within the ten largest alcohol companies operating in Ireland since 2010. We sought to address three questions: (i) what are the technological capacities of smart labels? (ii) what justifications does the alcohol industry use to normalise smart labels? (iii) how does this technology impact health when considered through a commercial determinants of health lens?

### Labelling and the shift towards smart labels

Labelling is a systemic part of product infrastructures [27]. It is regulated to protect the public right to informed choice and non-misleading information [27–30]. This is pertinent in the alcohol, ultra-processed food, and tobacco industries which have historically promoted consumption and perpetuated industry-favourable (mis)information through its labelling power [23, 31, 32]. Through their persuasive potential, brand-building power and visibility to consumers, labels are a key part of commercial marketing strategies [1, 3, 23]. Labelling regulation has been used as a relatively low-cost, yet effective population-level health intervention, for example, through standardised tobacco labelling, and amplification of health warnings on tobacco labels [33–35]. Increasingly, smart labels are used by transnational industries, from tobacco [36, 37] to ultra processed food [38–42] and alcohol [43, 44]. While under-explored within policy contexts, Trimble and colleagues show that Chinese tobacco companies use smart labels to proliferate consumption, direct consumers to promotional content, and circumvent public-health legislation [45]. A recent meta-review of alcohol health warnings (in various forms) concludes they the potential to significantly lower consumption and reduce alcohol sales [46]. Such findings pose a serious threat to the alcohol industry, and requires it to innovate to leapfrog potential public health policy around labelling.

In response to the increasing public health focus on product labels as potential sites for regulatory intervention, the alcohol industry deploys tactics to disrupt or circumvent new regulation on packaging. This includes misrepresenting evidence on the effectiveness of packaging measures to the government [47], venue-shifting to other areas of governance where more opposition to legislation might exist, or proposing voluntary action instead, even when evidence shows the ineffectiveness and low compliance to voluntary industry action on labels [1]. Various industries’ strong opposition to labelling regulation show how labels are a battleground where policymakers aim to use it as a platform to provide evidence-based health messaging, while industries seek to use the label to promote their product and emphasise the benefits of consuming it. As an increasing number of governments seek to implement measures on packaging, UCIs are tethering physical labels with digital capacities. The definition [48], legal interpretation [49], and conceptual understanding [50] of a “label” has remained unchanged since the 15th century; It has been interpreted as “information, *on* the packaging of a product” [48], also known as traditional front-of packet labelling [33]. However, technology is rapidly innovating what a “label” is and what its capacities are [50, 51].

**Fig. 2:**
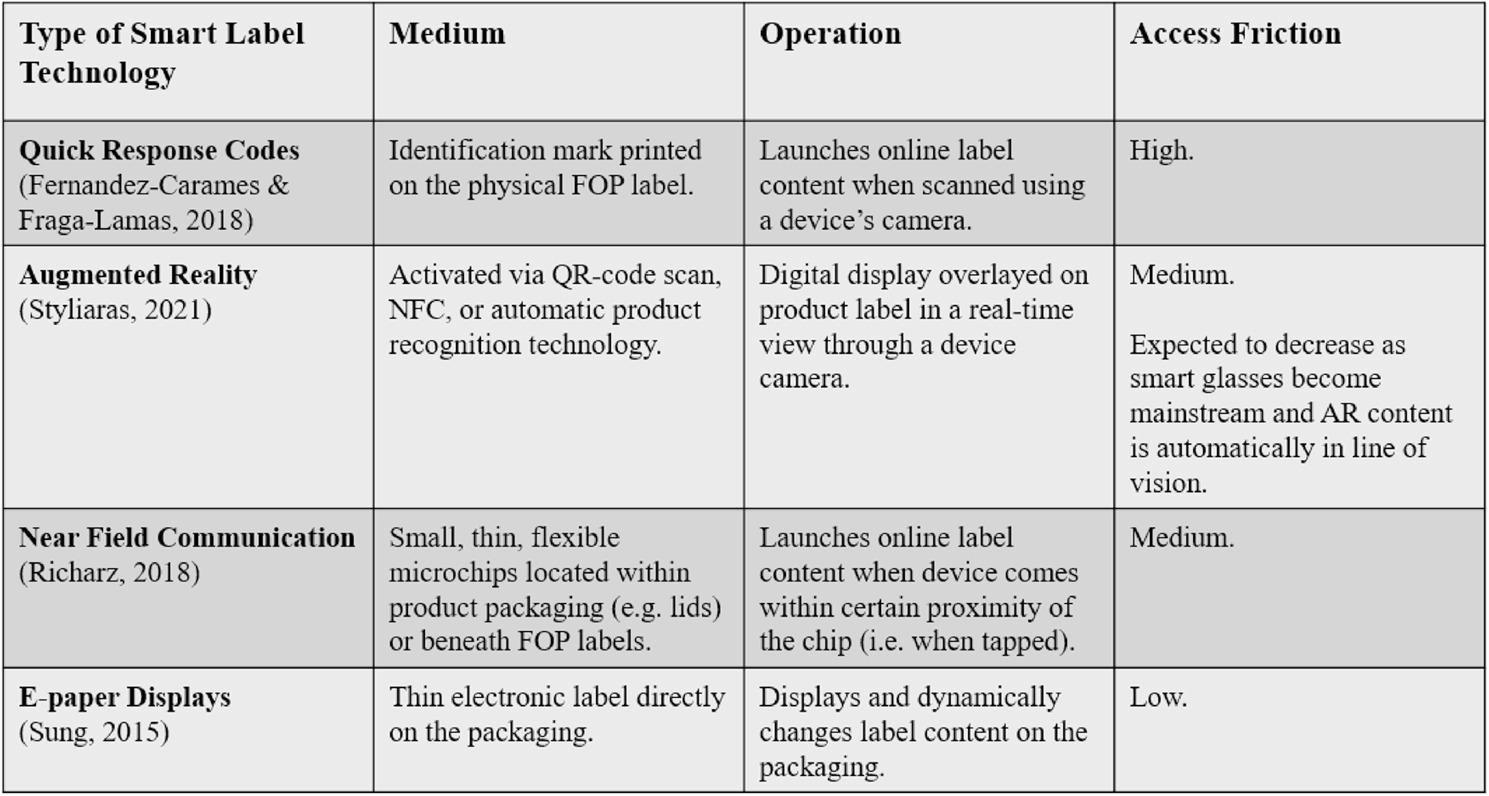
Explanation of selection of current smart label technologies

**Fig. 3:**
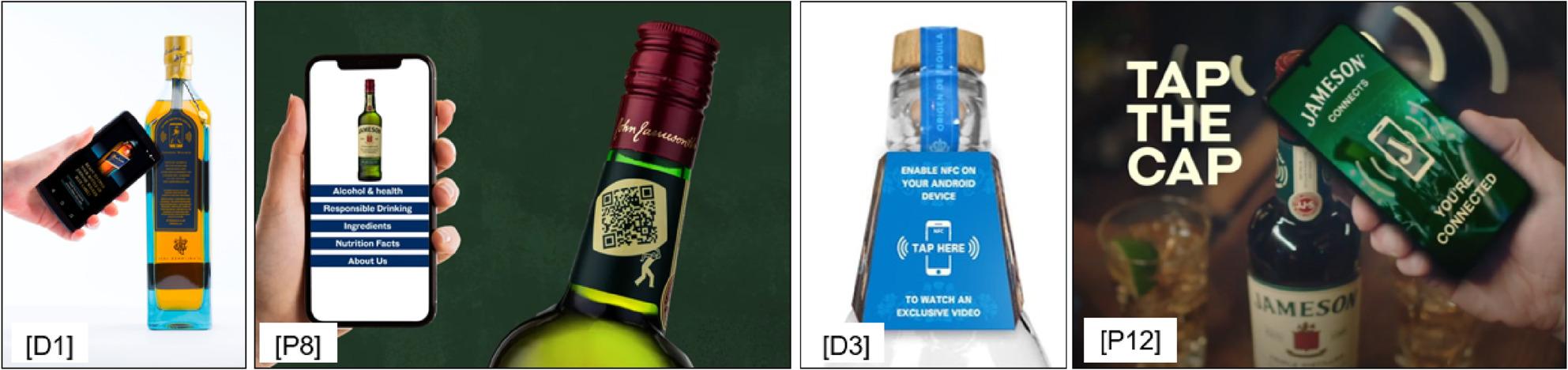
Smart labels. Example from this study's research domain

Smart labels are physical product features that function as gateways to digital content. These gateways take four principal forms: quick-response (QR) codes, near-field communication tags (NFC), augmented reality (AR) and e-paper displays (see Figs. 2 and 3). Using a device like a smartphone or smart glasses allows consumers to place the label in the line of vision of a camera, scan, or tap to expand and/or overlay traditional labels with expanded display real estate. For example, Corona used a QR-code-activated augmented reality experience that, when scanned via a smartphone, overlaid the physical label with an immersive virtual beach environment that offered exclusive recipes, integrated e-commerce functions, and social-media-ready interactive content [52].

Smart labels are often seen in industries as a requisite part of “Industry 4.0”, a new wave of industrial innovation where products, information, and communication are intelligently interconnected. Smart labels also have supply side capabilities, specifically consumer-tracking and “low-friction” product-reordering. Lowering friction covers many different ways to make the circuit between the production and the consumption of a product as seamless as possible, by one-tap ordering, faster loading times for images, or emphasising low commitment in form-filling [53]. Traditional labels present static, uniform content to a universal set which allow regulators to comprehensively and transparently review product claims. In contrast, smart label content is ephemeral, dynamic, and personalised, evading regulatory grasp.

### Irish alcohol labelling policy

Smart labelling is currently unregulated because the regulatory definition of a ‘label’ or ‘labelling’ is usually limited to the physical container. For example, in the Public-Health (Alcohol) Act 2018, in Ireland, a label is defined in the context of “labelling of alcohol products … *on* alcohol product containers” [54]. Similarly, food labels in the ‘European Union Provision of Food Information to Consumers Regulations 2014’ are defined as “*on* or *attached to* the packaging or container” [55]. In contrast, smart labels exist digitally, rather than physically. Despite this shift in labelling innovation, three assumptions currently prevent inquiry into smart labels as an priority action area for public policy: that there is low interest in accessing digital labels, particularly amongst older demographics, that consumers lack smart devices capable of accessing smart labels, and that information in smart labels falls secondary to traditional labels [33, 56, 57].

However, while policy currently assumes smart labels as a low threat to public-health, industry considers them a springboard. Smart labelling types are nascent; QR, AR, and NFC represent early-stage types through which smart labelling impact can be achieved, with lower-friction mechanisms such as smart glasses on the horizon [58]. Moreover, statistical information on which the above assumptions rest have become rapidly obsolete in this evolving area. While previous studies highlighted the lack of QR-scanner software possessed by consumers [57], this is now an inbuilt function on most mobile-devices [59]. In addition, as technology advances, the capabilities of smart labels exceed information provision. They present UCIs with three key capabilities. First, they transcend physical label boundaries [60], and are more akin to brand-owned entertainment channels [58, 61]. Without the restriction of physical, traditional label space, smart labels can alter policy-compliant traditional labels with alternative or additional information on nutritional, ingredient origins, and marketing campaigns [58, 62]. In this way digital labelling is distinct from marketing but can include it [50]. Secondly, smart labels not only impart but also collect information. They may contain sensors which capture real-time information on product usage, condition, and location, as well as the label’s performance in influencing these factors [6, 51, 60]. This collected consumer data is communicated back to businesses which in turn can remotely, dynamically, and automatically update the presentation of the label [6, 60, 61]. Thirdly, smart labels provide transient dynamic displays. The interactive experience of the smart label communicates to consumers at point-of-purchase as a traditional label would, but they can also uniquely communicate new and additional messaging post-purchase [51, 58, 62]. As a smart label gathers more data, it can become autonomous, using data-algorithms to personalise and predict consumer behaviour [60]. For example, Diageo used smart labels on Johnnie Walker Whisky bottles to send personalised marketing messaging to each user, a feature they advertised as a “premier digital experience” [63].

UCIs leverage rapidly evolving technologies to shift traditionally regulated paradigms like labelling. Regulation like the Public-health (Alcohol) Act 2018, formed within the preceding labelling-paradigm, now risks becoming insufficient, once again opening a window for the alcohol industry to leapfrog regulation. In this study, we measure the extent of smart labelling in the alcohol industry in Ireland, and detail the justifications the alcohol industry uses to normalise smart labels.

## Methods

In order to measure the prevalence of smart labels in Ireland, the top ten alcohol companies in Ireland were identified based on data from Euromonitor International (2024), reflecting market dominance and relevance to population-level exposure. Collectively, the brands within these companies represent over 70% of total alcohol sales in Ireland, selling over 380 million litres of alcohol in 2023 [64]. Alcohol Action Ireland estimates that this corresponds to 284 cans of beer, 12 bottles of spirits, 43 bottles of wine and 35 cans of cider per alcohol drinker [65].

We conducted our analysis in three parts. Our first goal was to identify smart label uses within the top ten companies in Ireland. We ran keyword searches under the keywords ‘smart-labelling’, ‘digital labelling’, ‘intelligent labelling’, ‘NFT’, ‘AR’, and ‘QR’ in 4 publicly available sources: the alcohol brand or company website or press centre, (ii) the smart labelling agency website or press centre (iii) 14 trade magazines across the alcohol, retail, marketing, and packaging sectors which have broad industry readerships, and (iv) 8 technology websites that focus on labelling innovations. If examples arose organically from these sources, which referenced another alcohol smart label that was in the top ten alcohol companies in Ireland, we followed it and included it.

The second part of our analysis examined the framing of smart labels within the alcohol industry. Framing theory guided our analysis. Research in the commercial determinants of health has made extensive use of framing theory, which focuses on how narratives are crafted via the calculated emphasis on, or exclusion of information [66]. Framing is an established mechanism used extensively by the alcohol industry to influence health policy [67, 68]. Specifically, Savell, Fooks, & Gilmore’s [68] systematic review of frames used by the alcohol industry guided the analysis, by examining how the industry laid out the multi-benefits of smart labelling and demonstrated evidence on its effectiveness. We collected information on the technological capability, the evidence for the effectiveness of the technology, and the perceived advantages from the industry standpoint. We identified 5 recurring frames that position smart labels as powerful for the alcohol industry.

Thirdly, we applied a commercial determinants of health lens to explore how smart labelling technologies may function to harm public health. Drawing on the approaches of Ulucanlar et al. [69], we critically reviewed the dataset and identified four key recurring patterns through which smart labels frame the narratives and mechanics of alcohol consumption in ways which harm public health and undermine policy.

The full data set and our coding framework are available at OSF: https://osf.io/5c64q/ [70]. Cases discussed in our results are referenced in square brackets with an ID tag corresponding to where it is in the dataset (e.g. [C2]).

## Results

### The prevalence of smart labelling in the alcohol industry (Ireland)

**Fig. 4:**
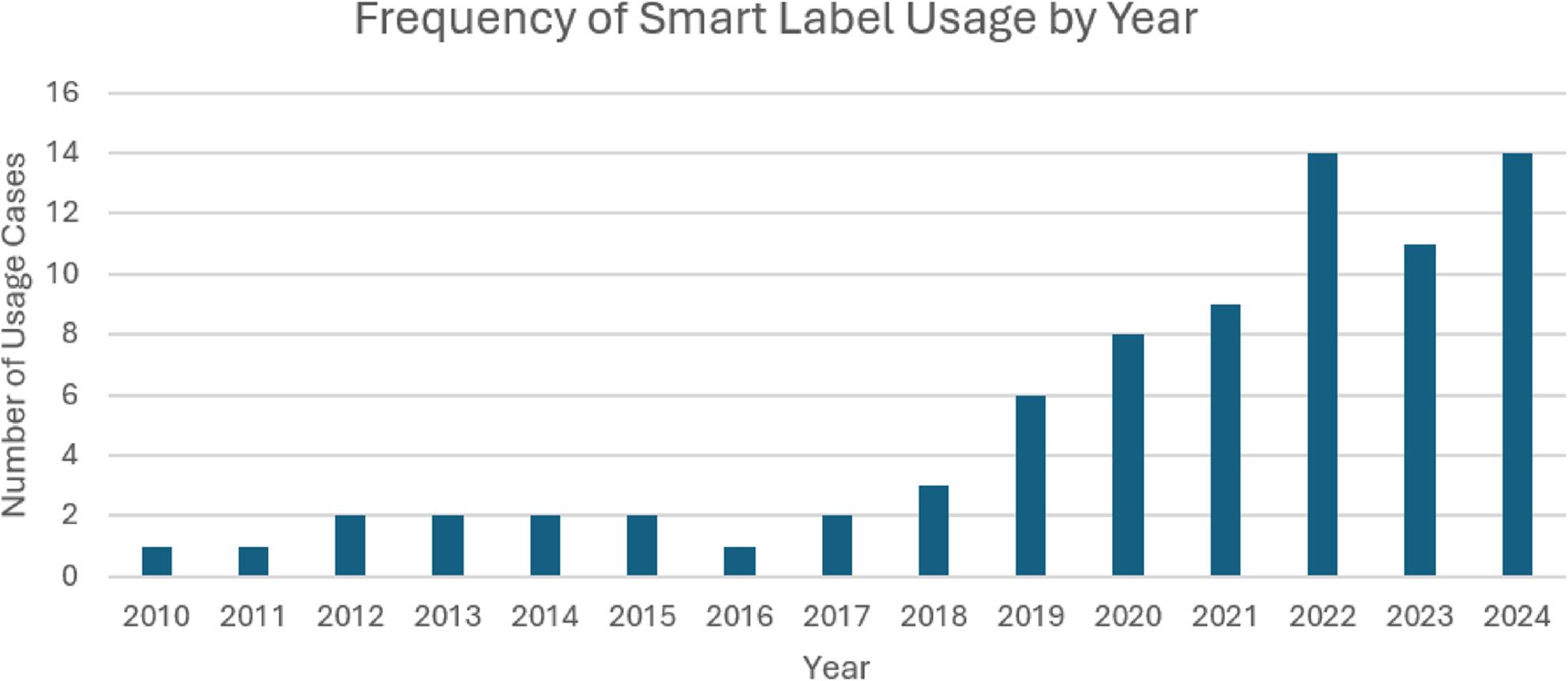
Frequency of smart label usage by year

Eighty-three cases of smart labels across forty-one distinct alcohol brands were identified. Nine out of the top ten alcohol companies in Ireland have used smart labelling to promote their brands. Predominant types of smart labels were QR-code (50%), AR (38%), and NFC (12%) technology in the smart labels observed. Smart label technologies began with low usage frequency in the early 2010’s. Frequency notably increased after 2018, correlating with technological advancements in smartphones whereby QR-scanning became standard for smartphone cameras [71] (See Fig. 4).

### Alcohol industry framing of smart labelling

**Fig. 5:**
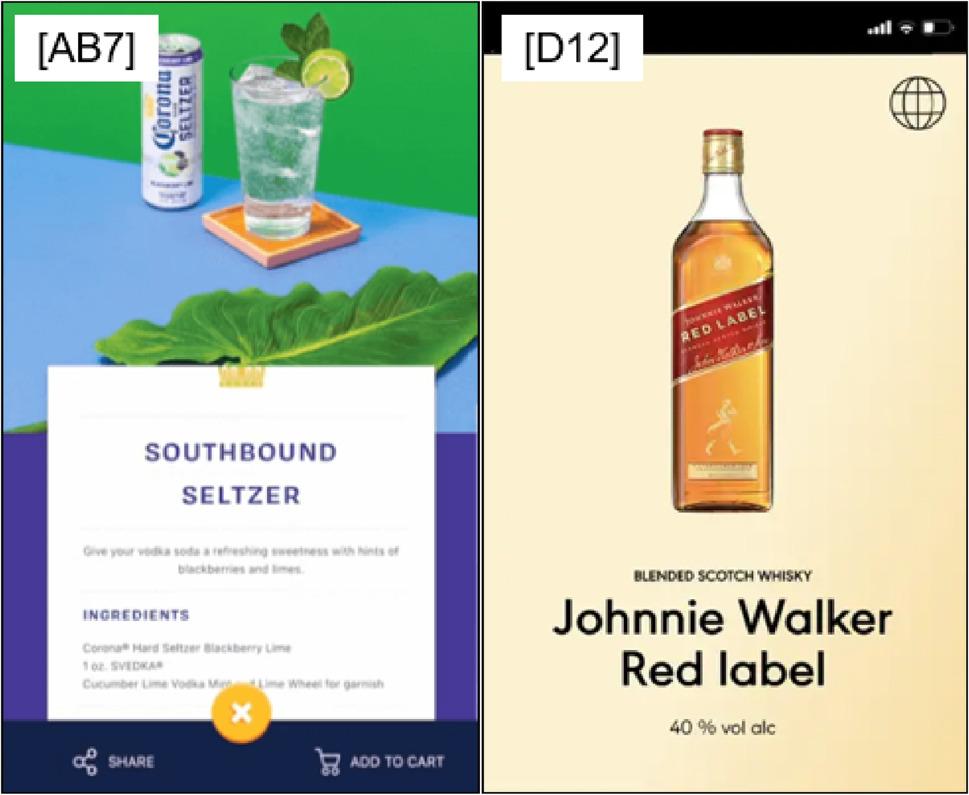
Smart labels present alternative nutrition information alongside marketing elements and repurchase opportunities

The alcohol industry produced five distinct frames of smart labels within the data, where the focus was on how smart labelling could produce positive outcomes for society, could empower the consumer, and could be a public health action – a win-win for the industry. We present it as a MORAL set of frames (Fig. 5; Table 1). Not all of the companies used these MORAL frames; some were solely focussed on how the technology can grow the size of the industry, as we detail in Section Understanding smart labelling through a commercial determinants of health lens.

**Table 1:**
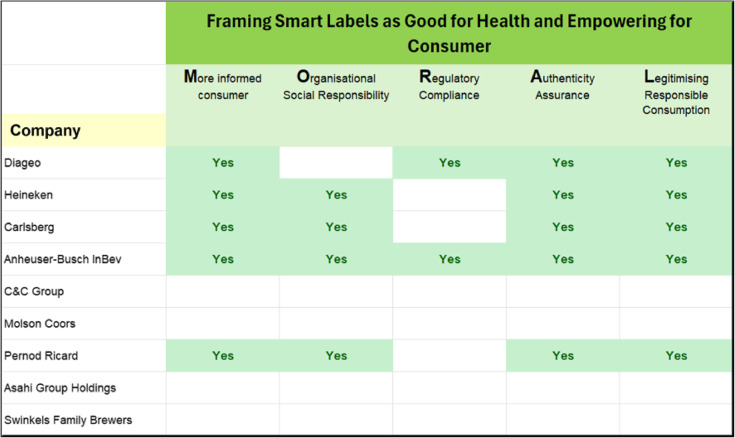
Usage of smart labelling by the alcohol industry

### M. more informed consumer frame

Smart labels are framed as a means of information-provision – the solution to uninformed consumption. Twenty-five such examples were observed within the data, used by five companies. This framing foregrounds a narrative that smart labels are policy aids, while backgrounding their capacity for policy circumvention. Industry actors argued that traditional labels provided insufficient space in comparison to smart labels which could provide consumers with “*unlimited information*” [AB9]. We found that when smart labels gave health advice, it was framed as a commitment to more-informed consumers. For example, Diageo framed disclaimers of responsibility as consumer-empowerment:

*“Our commitment … ensures that consumers … have all the information they need … to make educated choices when deciding to enjoy our brands.”* [D12].

*“By opening up as much information to you*,* our drinkers*,* as possible*,* we feel you’ll be armed to make the most informed and responsible decisions around your consumption of Jameson as possible. Sláinte!”.* [P8]

The more-informed-consumer frame serves to further blur the boundary between information and responsibility, shaping norms at a systemic level.

### O. organisational social responsibility

This study found twenty-three examples across four companies where smart labelling was framed as a platform for sustainability and philanthropy efforts. Anheuser-Busch InBev used Budweiser smart labels to allow consumers to vote for which US States would receive portions of their education scholarship to military families [AB2]. This emotion-based appeal demonstrates the framing of smart labels as a facilitator of organisational social responsibility. Other companies have used smart labels to highlight sustainability and inclusion initiatives:

*“Finding out the environmental footprint of your beer bottle by just one click? "* [H3].

*“Users can simply scan the Heineken label … and access the brand’s 2014 Sustainability Report*.*”* [H5].

UCI CSR functions as an indirect lobbying tactic to improve their public-image [72]. By portraying the alcohol industry as a problem-solver (of social inequality, discrimination and climate crisis) rather than a problem creator (of health-harm), smart labels aid the framing of the alcohol industry as a mechanism for social and ecological good.

### R. regulatory compliance

In a limited number of cases, the alcohol industry framed smart labels as supporting regulation, and as a means of complementing FOP regulation. Anheuser-Busch InBev framed smart labels as a step towards regulatory compliance: press releases highlighted that its smart label supplier was introduced to the company through a European Commission innovation initiative. While the European Commission actively works to reduce alcohol health harm, this does not preclude sub-agencies like the European Innovation Council working with alcohol companies. As a result, Anheuser-Busch InBev highlighted this connection, framing smart labels as an initiative supported by regulatory bodies [AB6]. When releasing smart labels on Bulleit Bourbon, Diageo framed the initiative as a means of respecting and even enhancing front-of-pack regulation, while at the same time enabling the delivery of branding messages to consumers [D3].

### A. authenticity assurance

This study noted thirty-eight examples across five companies in which smart labels were framed as a mechanism of assuring product authenticity. Companies claimed that smart labels enhanced product-authenticity and tamper-protection: For example, Heineken’s “Hop on the Blockchain” [H3], Budweiser’s “Track Your Bud” [AB8], and Martell’s “Bottle Identification and Verification” [P5]. This frame utilises the language of transparency to frame smart labels as a technology of quality assurance.

### L. legitimising responsible consumption

Twenty-one examples across five companies were observed where smart labels were framed as a means for promoting responsible consumption. The alcohol industry portrayed smart labelling as a proactive strategy of encouraging responsible consumption. For example, the Carlsberg framing of their smart label use reads: “*Digital transformation is about so much more than creating a commercial boost - it also has the power to promoting* [sic] *responsible drinking habits and choices at a massive scale*” [C2].

*“Irish Distillers… has launched a digital e-label system which will better inform consumers …*,* as well as encourage responsible drinking*.*”* [P1].

*“Helping to make moderate drinking aspirational and demonstrating respect and truthfulness in all our activations.”* [H1].

## Understanding smart labelling through a commercial determinants of health lens

Drawing on a commercial determinants of health perspective we re-analysed the 83 smart labelling cases to examine how smart label framing strategies may contribute to ill health. We find that smart labels deploy consumer-centred, health-centred, and health-policy-centred frames to habituate consumption, provide alternative forms of information that undermine public health, enable targeting of consumers, and mould industry norms. In doing so, these frames systematically redirect attention away from underlying industry practices – specifically industry expansion, data-driven consumer targeting, and regulatory avoidance. We bring these dynamics together under what we term the H.A.R.M. framework (Table 2), the frames of which we discuss below.

**Table 2:**
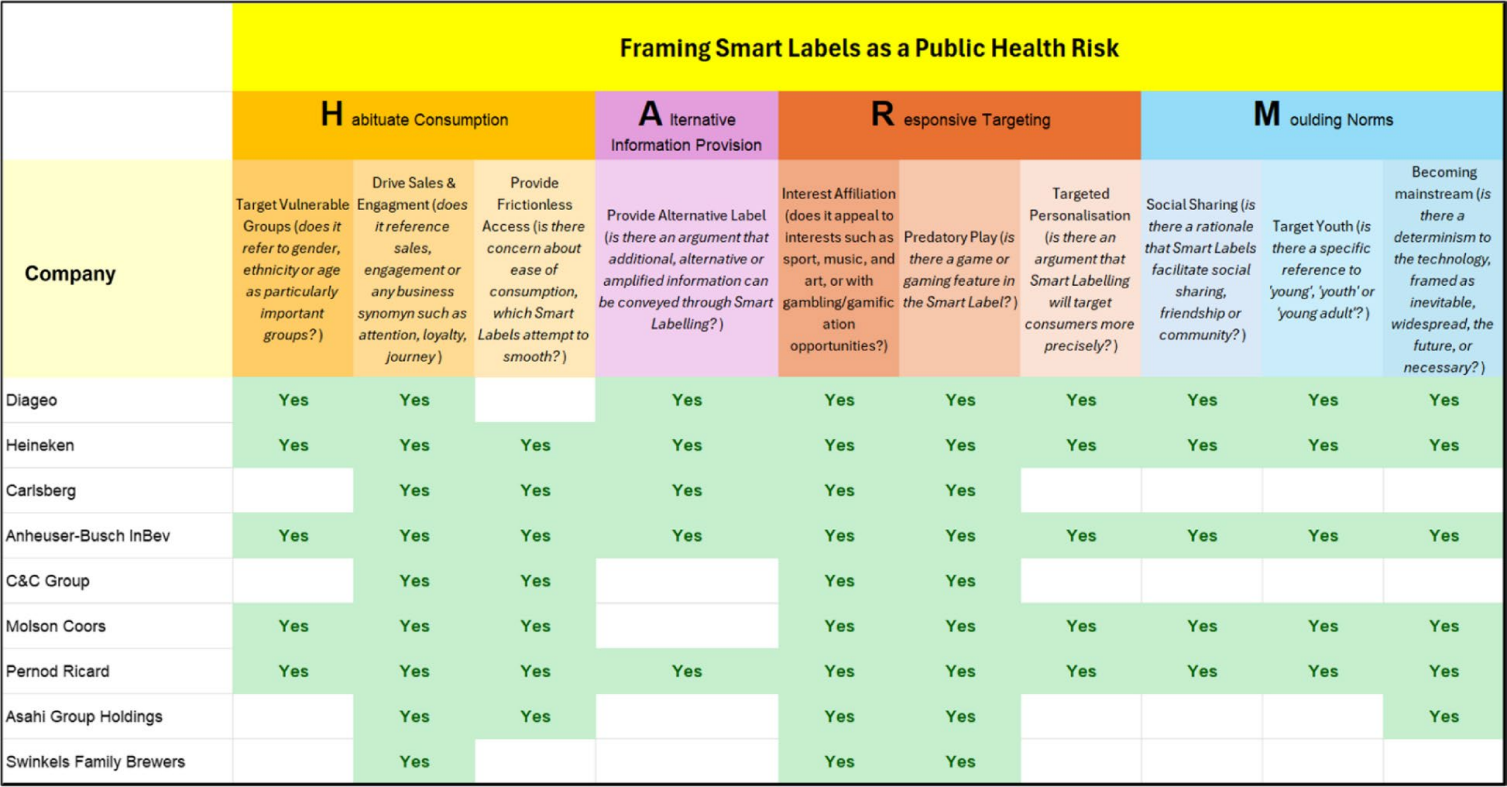
Usage of smart labelling by the alcohol industry

### H. habituate consumption

All nine companies identified as smart label users in this study used smart labelling to habituate consumption, driving both general sales and repeat purchases for the general population. For example, Pernod Ricard’s press release for ‘Jameson Connect’ cited smart label’s “persuasive content” [P8] as a driver of consumption which encourages repeat purchases.

*“Helping Budweiser … ultimately drive purchases in store or through the e-commerce.”* [AB3].

*“Bulk products are generally more pricey than a single can … However*,* by attaching the QR code on the packaging of the bulk product itself*,* the brand managed to significantly make it more appealing.”* [H6].

Regulation often introduces “access-friction” to environments, like licensing hours, which interrupt habitual alcohol consumption. However smart labels have the potential to circumvent this. 7 of 10 alcohol companies framed smart labels as explicitly reducing friction to access. For example, the Tequila brand OTACA leverages frictionless access by providing instant reordering opportunities through their smart labels on its bottles [73].

*“The process is really simple and as smooth as the liquid in the bottles; just scan the QR-code on the Jameson bottle*” [P8].

Additionally, technologies are now evolving to increase accessibility to smart labels, furthering the reach of habituation-enabling features. For example, Heineken transitioned from app-based to ‘LogoGrab’ technology, thereby removing the need for consumers to scan QR-codes or download brand-owned apps to access the smart label.

Smart labels were also found to be used by 5 of the 10 companies to increase regular consumption amongst younger demographics. 6 of the companies specifically referenced the “young”, “social” and “tech-savvy” digital natives whom they aim to engage and retain with smart labels. Although alcohol brands in our dataset claimed to target college-students and Gen Z/Millennial generations, smart label strategies include features that appeal to broader youth demographics, including children. Smart labels on alcohol that feature gaming mirror the most popular applications of augmented reality (AR) applications to date. For example, Pernod Ricard created an interactive AR environment similar to Pokémon Go [74] – a game designed for children. However, instead of simulating the collection of mythical creatures, it simulates the consumption of Absolut Vodka:

*“The virtual experience is complete with a stage*,* special effects and within the virtual setting*,* people even have the chance to order Absolut cocktails at the bar.”* [P3].

By establishing a connection with young people via smart labels, alcohol brands can foster lifelong drinking habits [75]. A trade magazine entry ([P11]) highlights how Pernod-Ricard recognised that young people have concerns about alcohol health-harms, then used smart labels to divert attention from health-harm messages to marketing messages by gamifying an AR-guided whiskey-tasting:

*“Younger adults tend to drink less beer because of health concerns*,* and when they do drink*,* they seek out diversity in their alcohol choices . Whisky is well suited to this kind of exploration”* [P11].

### A. alternative information provision

Smart labels can present alternative nutrition information alongside marketing elements and repurchase opportunities, undermining the regulated information that is required on physical alcohol labels (See Fig. 5).

**Fig. 6:**
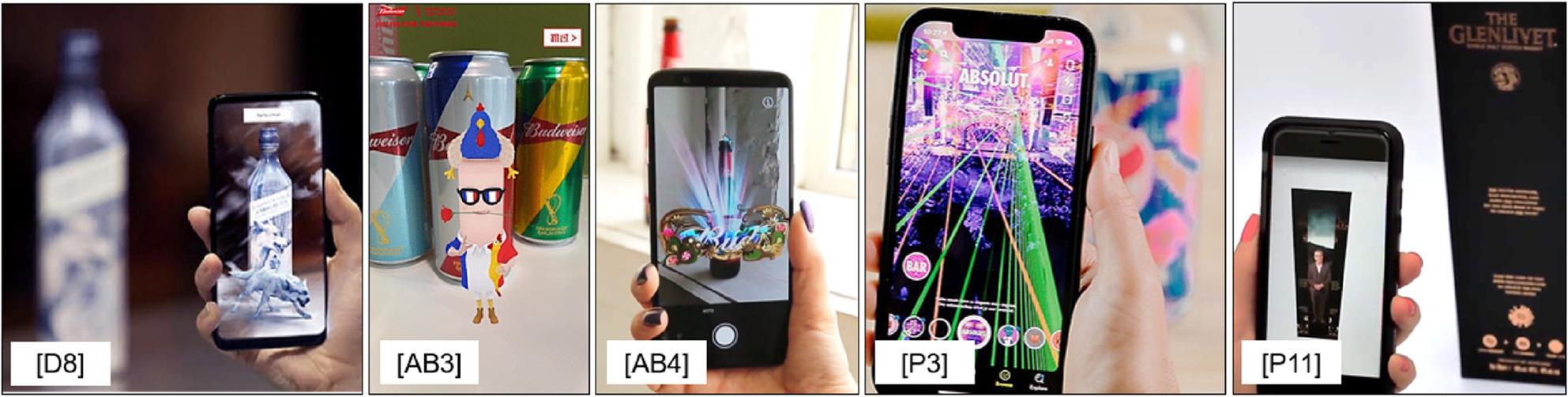
Smart labels overlay physical labelling and health warnings with marketing elements

This study found 17% of smart labels provided an alternative nutritional/warning label. These alternative nutrition/warning labels contained a mix of product information and marketing elements: Diageo, Anheuser-Busch InBev, and Pernod Ricard smart labels presented nutritional information and responsible consumption guidelines alongside cocktail recipes and tasting-note characteristics. The integration of health and marketing information diminishes the visibility and clarity of mandated health warnings (See Fig. 6). In the attention economy, traditional labels are forced to compete with smart labels [28]. Evidence on traditional labels support this, showing that bias towards secondary information consumes the finite cognitive effort allocated to reading product labels [34]. This concurs with the work of Newall [76] and Petticrew [77] regarding the steering effects in corporate social responsibility messages.

### R. responsive targeting

Smart labels were found to be a prime example whereby intelligent capabilities are used to target consumers via (i) personalisation, and (ii) interest affiliation and predatory play.

Five companies within the data provided personalised smart labels to consumers, and three outlined data-collection techniques in their strategy materials, describing how the technology enabled them to track how the product is purchased, interacted with, consumed, repurchased, as well as its temporal and geographical location. These data were then used to deliver personalised label content, which changed dynamically to optimise engagement. Smart label data collection provides the alcohol industry with detailed insights into consumption behaviours and demographics. For example, Pernod Ricard used NFC labels to monitor purchase patterns, consumption behaviour, and location to provide consumers with localised marketing content and geo-located nearby bars selling Malibu rum [P4]. Within trade magazines, this was identified as a way the industry could deploy smart labelling to achieve its full potential [58, 61, 78]. Yet the extent to which customers are aware of this data-collection is unknown.

Responsive targeting was also found in the form of interest affiliation and predatory play. Across all eighty-three -cases, 59% (n = 49) facilitated gaming or gambling, a strategy used by nine out of ten alcohol companies. Of the 44 cases which affiliated the product with consumer interests such as sport, music, and art, 24 also presented consumers with gambling/gamification opportunities.

*“Budweiser launched QR-codes for fans to win free world cup tickets and one year’s supply of Budweiser beer.”* [AB9].

*“The $62*,*400 cash prize could buy a Heineken 12-bottle pack every week for 60 years.”* [H13].

*“Incredibly*,* every ‘1 in 10’ entry will be successful*,* and winners can redeem their complimentary Bulmers on the spot”* [H8].

### M. moulding industry norms

Six of nine companies actively sought to establish smart labels as an expected industry practice and socially accepted norm. This study found twenty-nine cases of the alcohol industry establishing the expectation for alcohol labels to be smart labels. Trade magazines call on other companies to “*get on board now*” [H4], use the “*always-on consumer presence*” [P4] and start “*creating real-time 3D assets*” [M7].

20% of cases facilitated social sharing of smart label content or interaction. Heineken, AB-InBev, and Pernod Ricard smart labels facilitated exclusive selfies to be shared on social media. Diageo, AB-InBev, Molson-Coors, and Pernod Ricard prompted smart label game-scores to be shared. The label’s ability to facilitate social sharing frames alcohol consumption as a fun, recreational activity. In addition, the alcohol industry capitalises on the digital domain of smart labels to enter social media conversations. Whether branded selfies or game scores, the purpose of prompting users to share content from smart labels is to establish smart labelling as an expected industry practice. This digital redistribution of social material by the public is beyond the remit of traditional policy considerations [79]. By facilitating social sharing via smart labels, alcohol brands embed social labels as a normal industry practice that is pervasive. This in turn makes it more difficult for policy makers to legislate against it. Norms become underlying drivers for CDoH vectors [4]. The norm-establishment of smart labels may amplify the scale at which the alcohol industry can habituate consumption, provide alternative labels, and responsively target consumers.

## Discussion: a digital vector of disease

Through analysing the use of smart labels by the Irish alcohol industry through the lenses of both the industry framing and the commercial determinants of health, this study has highlighted the strategic positioning of smart labels by industry actors as technologically innovative and consumer-friendly, even though the content and form of the labels themselves may drive further population harm. Through introducing the H.A.R.M framework, this study has demonstrated how smart labels harm public health. Importantly, the alcohol industry’s strategic framing of smart labels, categorised according to the MORAL framework, indicates that these consequences are not incidental but rather constitute a tactic to circumvent regulation under the guise of technological innovation. The health effects from the smart labelling of alcohol are obfuscated by industry narratives that emphasise ‘responsible consumption’, co-opt the language of transparency, and maintain that the focal concern is the user experience. Understanding context-aware, dynamic and personalised capabilities of smart labelling is crucial, given the prevalent adoption of smart labelling amongst a breadth of UCIs which are subject to regulation of their traditional labels.

Smart labels are framed as a means of information-provision – the “solution” to uninformed consumption. Twenty-five such examples across five companies were observed within the data set. This framing by the alcohol industry establishes an assumption that smart labels are policy *aids*, while obscuring their capacity for policy circumvention.

Existing labelling definitions that are often used in regulation are increasingly inadequate as labelling-digitalisation reshapes regulatory definitions and assumptions at rapid pace. It is not the framing of smart labels in isolation which are shifting labelling norms, but also the rapid technology innovation itself. The findings of this paper should therefore be read in the context of increasing use of smart labels, which are on a trajectory for mass-prevalence and application in packaged goods. Their capabilities are increasing, while size, material rigidity and cost decrease. Myny (2018) notes that the microchips used for smart labels, like NFC tags, follow Moore’s Law [80] whereby the number of transistors on the microchip doubles every two years. Similarly, the substrates required to hold microchips are rapidly evolving, resulting in smaller, lighter, and more powerful computing abilities at lower cost [81, 82].

**Fig. 7:**
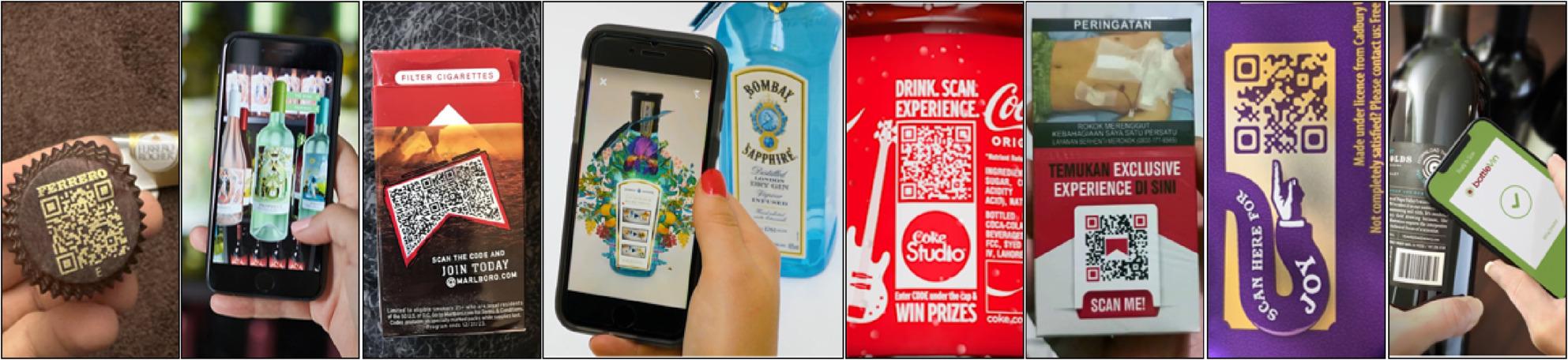
Smart labelling across unhealthy commodity industries

Smart labelling is forecast to value $17.33 billion by 2029 [83], demonstrating the scale of uptake in consumer markets (Fig. 7). This and other studies have similarly found that smart labelling uptake has particularly increased since the pandemic; NFC-payment methods, QR-code based contact-tracing, and remote ordering accelerated contactless scanning/tapping behaviours, normalising digital methods of interaction with, and payment for, products [58, 84, 85]. With its prevalence increasing, it is imperative that health policy addresses smart labels in regulation. While specific smart labelling technology will change over time, it is the primary logic: to habituate consumption, to provide alternative information that is entertaining and distracting, to respond to consumers interactively in real time, and to pre-empt regulation by moulding norms which will remain as the key strategic outcomes of the alcohol industry. It is these implications of the policy-digitalisation gap, illuminated by the H.A.R.M. framework, which merit policy consideration.

### Limitations

This study has limitation which should be considered.

Firstly, this research generated the dataset abductively, meaning we searched for press releases on smart label innovations on the company website in a top-down manner, and then allowed this to snowball organically out to trade magazines that specialised in packaging technology, drinks’ industry magazines, and advertising agencies that featured smart labelling case studies on alcohol brands. Future research could approach the collection differently, by for example, examining all drinks’ industry magazines in a given region systematically. Further, we did not examine products physically in retail settings, and it is possible that alcohol products in Ireland have smart labels but are not announced on any of the four types of data sources we searched under.

Secondly, this study did not seek to evaluate or propose specific regulatory or policy solutions. The focus was deliberately concerned with identifying and characterising smart labelling practices, analysing their industry framing, and how they may function as a commercial determinant of health. While the findings have clear regulatory relevance, policy solution proposal falls outside the scope of this paper and represents a key area for future research.

Third, although the research highlights that smart labels enable data collection and personalised, responsive targeting, it acknowledges that the extent to which customers are aware of these practices, understand how their data is used, or perceive associated risks is unknown. Further qualitative study is needed to examine consumer understanding of data-collection by smart labels on alcohol products.

Finally, this research was restricted to publicly available sources. As a result, the findings may underestimate the full scope and sophistication of smart label usage by the alcohol industry.

## Conclusion

This study has examined a case of a rapidly advancing, consumer-focussed technology which poses a threat to public health. By categorising the impact of smart labels via two perspectives – the justifications of the alcohol industry, and a commercial determinant of health perspective. This harm occurs in the context of the policy-digitalisation gap, which enables the impacts to evade regulation. Smart labels habituate consumption, provide alternative information, enable responsive targeting, and mould industry norms, contributing to long-term increases in alcohol use and related harms. As such, smart labels may be conceptualised as a digital vector of disease and an emerging commercial determinant of health. Furthermore, these harms are actively concealed by industry narratives describing smart labels as promoting responsible consumption, providing transparency, or enhancing user experience. These findings underscore the expanded playbook capabilities of industry to outpace policy in the digital age and should be considered a call-to-action for policy to rapidly evolve or to perpetually remain one step behind.

## Data Availability

All data is available on the Open Science Framework: \The use of smart labels in the alcohol industry\ (Ireland). [Online].; 2025. Available from: [https://osf.io/5c64q/overview](https:/osf.io/5c64q/overview).

## Abbreviations

AC: Anna Coghlan
NC: Norah Campbell
AvA: Amber van den Akker
